# Severe Mental Illness Among Adults with Atopic Eczema or Psoriasis: Population-Based Matched Cohort Studies within UK Primary Care

**DOI:** 10.2147/CLEP.S384605

**Published:** 2023-03-17

**Authors:** Elizabeth I Adesanya, Alasdair D Henderson, Julian Matthewman, Ketaki Bhate, Joseph F Hayes, Amy Mulick, Rohini Mathur, Catherine Smith, Helena Carreira, Sujit D Rathod, Sinéad M Langan, Kathryn E Mansfield

**Affiliations:** 1 Department of Non-Communicable Disease Epidemiology, London School of Hygiene & Tropical Medicine, London, UK; 2 Division of Psychiatry, University College London, London, UK; 3 St John’s Institute of Dermatology, Guys and St Thomas’ Foundation Trust and King’s College London, London, UK; 4 Department of Population Health, London School of Hygiene & Tropical Medicine, London, UK

**Keywords:** epidemiology, dermatology, psychology

## Abstract

**Background:**

Existing research exploring associations between atopic eczema (AE) or psoriasis, and severe mental illness (SMI – ie, schizophrenia, bipolar disorder, other psychoses) is limited, with longitudinal evidence particularly scarce. Therefore, temporal directions of associations are unclear. We aimed to investigate associations between AE or psoriasis and incident SMI among adults.

**Methods:**

We conducted matched cohort studies using primary care electronic health records (January 1997 to January 2020) from the UK Clinical Practice Research Datalink GOLD. We identified two cohorts: 1) adults (≥18 years) with and without AE and 2) adults with and without psoriasis. We matched (on age, sex, general practice) adults with AE or psoriasis with up to five adults without. We used Cox regression, stratified by matched set, to estimate hazard ratios (HRs) comparing incident SMI among adults with and without AE or psoriasis.

**Results:**

We identified 1,023,232 adults with AE and 4,908,059 without, and 363,210 with psoriasis and 1,801,875 without. After adjusting for matching variables (age, sex, general practice) and potential confounders (deprivation, calendar period) both AE and psoriasis were associated with at least a 17% increased hazard of SMI (AE: HR=1.17,95% CI=1.12–1.22; psoriasis: HR=1.26,95% CI=1.18–1.35). After additionally adjusting for potential mediators (comorbidity burden, harmful alcohol use, smoking status, body mass index, and, in AE only, sleep problems and high-dose glucocorticoids), associations with SMI did not persist for AE (HR=0.98,95% CI=0.93–1.04), and were attenuated for psoriasis (HR=1.14,95% CI=1.05–1.23).

**Conclusion:**

Our findings suggest adults with AE or psoriasis are at increased risk of SMI compared to matched comparators. After adjusting for potential mediators, associations with SMI did not persist for AE, and were attenuated for psoriasis, suggesting that the increased risk may be explained by mediating factors (eg, sleep problems). Our research highlights the importance of monitoring mental health in adults with AE or psoriasis.

## Introduction

Atopic eczema (AE) and psoriasis are common inflammatory skin diseases associated with substantial morbidity and impaired quality of life for both sufferers and their families.^1^,^2^ Worldwide, AE affects 1–3% of adults, and psoriasis affects up to 2% of adults.^3^,^4^

Severe mental illnesses (SMIs, including schizophrenia, bipolar disorder, and other psychoses) are long-lasting psychological conditions affecting approximately 0.9% of the UK population.^5^ People with SMI experience substantial health inequalities including a higher prevalence of chronic comorbidities (eg, respiratory, or cardiovascular disease), a shorter life expectancy (up to 20 years) and increased mortality than the general population.^5^,^6^

Substantial evidence demonstrates associations between atopic eczema or psoriasis and several psychiatric comorbidities including depression,^7–10^ anxiety,^7^,^10^ and suicidality.^7–10^ Limited evidence from cross-sectional^11–13^ and case–control^14–17^ studies suggest an association between AE or psoriasis and SMIs. Potential mechanisms for the relationships are unclear; however, proposed hypotheses include shared genetic susceptibility, immune dysregulation, unhealthy lifestyle choices (eg, harmful alcohol use), and chronic itch in AE leading to sleep deprivation and subsequent psychiatric symptoms.^13^,^14^,^18–22^

Longitudinal evidence for associations between AE or psoriasis and SMI in adults are particularly scarce, with only a few studies – with important limitations (including small study populations or focus on specific SMIs) – aiming to address temporal associations.^23–27^ Temporality of associations between AE or psoriasis and SMI in adults are therefore unclear. Temporal associations between AE or psoriasis and SMI could substantially impact public health as AE and psoriasis are common, and there is considerable morbidity and mortality associated with SMI.

We undertook matched cohort studies using primary care electronic health record data to investigate longitudinal associations between AE or psoriasis, and incident SMI in adults. We also explored whether associations varied with AE or psoriasis severity.

## Methods

### Study Design and Setting

We conducted two matched cohort studies between January 2, 1997, and January 31, 2020, using primary care electronic health record data from the UK’s Clinical Practice Research Datalink (CPRD GOLD). CPRD is an ongoing, nationwide primary care database of routinely collected and anonymised medical records that includes approximately 7% of the UK population.^28^

### Study Population

All adults (≥18 years) with at least 1 year of registration with a general practice meeting CPRD quality-control standards during the study period were eligible for inclusion. We identified two matched cohorts: 1) people with and without AE and 2) people with and without psoriasis. We matched (without replacement) each adult with AE or psoriasis on age (within 5 years), sex, and general practice with up to five adults without AE (in the AE cohort) or psoriasis (in the psoriasis cohort) in calendar date order (Appendix S1). Adults with AE and psoriasis were identified using previously validated definitions.^29^,^30^ The AE definition required records of at least one diagnostic code, and at least two skin disease therapies (eg, phototherapy or prescriptions for topical or oral drugs) recorded on separate days. Individuals were determined as having AE from the latest record of either their first AE diagnostic code, or the second record for AE therapy. The psoriasis definition required a record of at least one diagnostic code.

**Figure 1:**
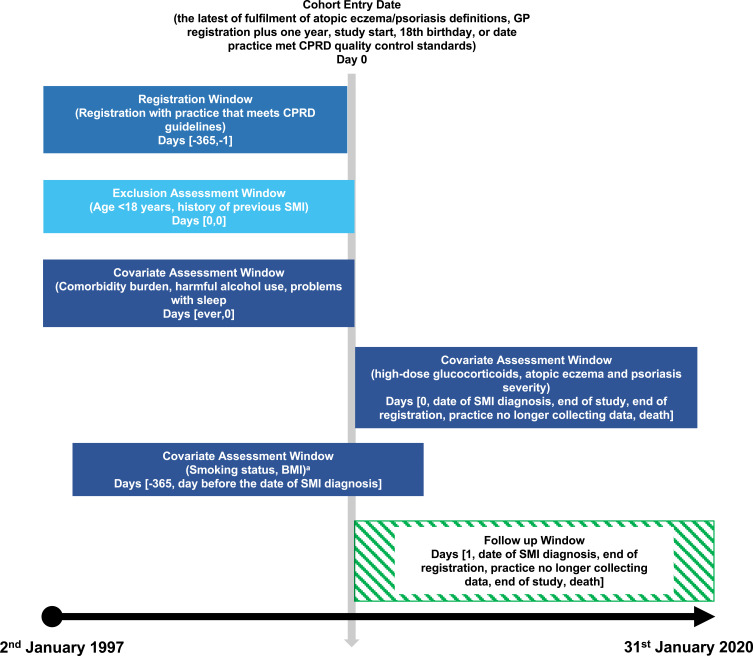
Diagram of cohort study design, describing the dates of cohort entry, covariate assessment, follow-up, and study exit. ^a^Identified using an algorithm where records identified within −1 year to +1 month of the index date are regarded as the best, +1 month to +1 year from the index date as second best, the nearest before −1 year from the index date as the third best, and the nearest after +1 year from the index date as the worst. Smoking status or body mass index recorded after severe mental illness diagnosis were not used.

**Abbreviations**: BMI, body mass index; CPRD, Clinical Practice Research Datalink; SMI, severe mental illness.

We followed individuals with AE or psoriasis from the latest of (index date): study start (January 2, 1997); date they met AE or psoriasis definitions; 1 year after the date of registration with their practice; date their practice met CPRD quality-control standards; or their 18th birthday. Follow-up for individuals without AE or psoriasis began on the same date as matched individual with AE or psoriasis. Follow-up ended at the earliest of SMI diagnosis; study end (January 31, 2020); end of registration with practice; practice no longer contributing data to CPRD; AE or psoriasis diagnosis (for adults without) or death (Figure 1).

### Outcome

We identified SMI based on the earliest primary care record of an SMI diagnostic Read code (schizophrenia, bipolar disorder, other non-organic psychoses). We considered broader definitions of SMI (including “symptom” codes, eg, delusions) in sensitivity analyses (Table S1). We excluded adults with a diagnosis of any SMI before follow-up began. Individuals with prior depression or anxiety were not excluded as they may represent early symptoms of SMI or be present in individuals later develop SMI.

### Covariates

We used directed acyclic graphs (DAGs) and a systematic review to inform covariate selection (Appendix S2).^31^,^32^ We matched on age, sex, and general practice, and considered calendar time (1997–2003, 2004–2009, 2010–2015, 2016–2020) and deprivation (quintiles of patient-level Carstairs Index used when available, otherwise practice-level Carstairs Index used) as potential confounders.^33^ We considered the following variables as potential mediators: comorbidity burden (using the Charlson Comorbidity Index),^34^ body mass index (BMI), smoking status, harmful alcohol use, and in AE only, sleep problems and high-dose glucocorticoid use. We considered age, sex, and calendar period as potential effect modifiers as associations between AE or psoriasis and SMI may be modified by age and sex differences in SMI prevalence,^5^ and changes in clinical, diagnostic, and administrative practices over time. Further details on covariate definitions are available in Appendix S3. Justifications of variable roles are available in Appendix S2.

### Statistical Analyses

#### Main Analysis

We initially described characteristics of adults with and without AE or psoriasis. We used Cox regression, stratified by matched set,^35^ with current age as the underlying timescale to estimate hazard ratios (HRs) and 95% confidence intervals for associations between AE or psoriasis, and SMI.

We initially constructed minimally adjusted models including only the main exposure variable (AE or psoriasis) and implicitly adjusting for age (through underlying timescale) and matching variables (age, sex, general practice) by stratifying on matched set, followed by sequential models further adjusting for other potential explanatory variables. In sequential models, we 1) adjusted for potential confounders (deprivation and calendar period) and 2) further adjusted for factors potentially on the causal pathway that mediate associations between AE or psoriasis and SMI (comorbidity burden, harmful alcohol use, smoking status, and BMI, and, in AE only, sleep problems and high-dose glucocorticoid use). Using sequential models allowed us to differentiate between the direct (eg, inflammatory effect of AE or psoriasis) and total effect through the skin disease and other potential mediating variables (eg, lifestyle factors) of AE or psoriasis on SMI. We assessed proportional hazards assumptions using Schoenfeld residuals (Appendix S4). All data were managed and analysed using Stata V16 (StataCorp, Texas, USA). Code lists for covariates and analysis code are available to download from an online repository.^36^

We estimated absolute incidence rates and rate differences for incident SMI in both AE and psoriasis cohorts. We repeated our analysis in a series of sensitivity analyses to assess the robustness of our findings (details in Table S1).

#### Secondary Analyses

We investigated associations between AE or psoriasis severity and by classifying individuals with AE as having mild, moderate, or severe disease, and individuals with psoriasis as having mild or moderate-to-severe disease using previously developed definitions.^37^,^38^ We updated severity over time, and compared SMI risk at each severity level with risk in those without AE or psoriasis using stratified Cox regression. We stratified analyses separately by age, sex, and calendar period to investigate whether they modified associations between AE or psoriasis and SMI. We used likelihood ratio tests to test for statistical evidence of effect modification. We individually tested effects of potential mediators on confounder-adjusted associations between AE or psoriasis and SMI in a post hoc analysis.^39^ Each potential mediator was added in a separate model. Further details on secondary analyses are available in Appendix S5.

## Results

### Baseline Characteristics

**Figure 2:**
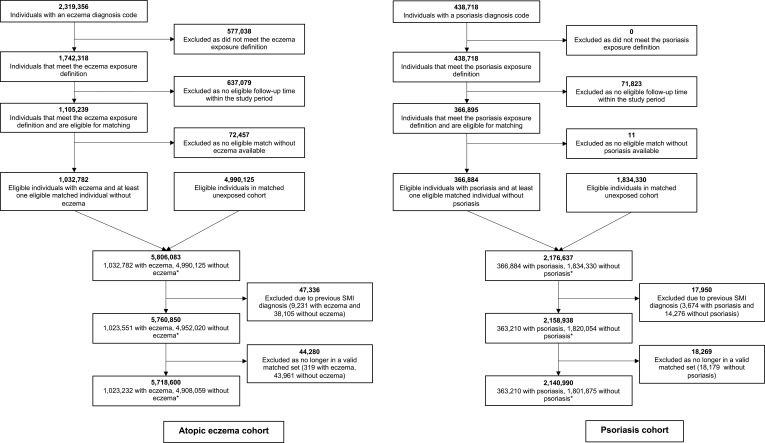
Flowchart illustrating identification of participants in atopic eczema and psoriasis cohorts. *Numbers of people with and without atopic eczema or psoriasis do not sum to the total number of individuals included in each cohort. Individuals with atopic eczema or psoriasis could be included in the matched comparison cohort up until the date of their first atopic eczema or psoriasis diagnosis.

We initially identified 1,032,782 adults with AE matched to 4,990,125 without, and 366,884 with psoriasis matched to 1,834,330 without who were eligible for study inclusion (Figure 2). After excluding those with SMI diagnosed on or before follow-up began, and those who were not in valid matched sets (including at least one exposed and one unexposed individual), 1,023,232 adults with AE matched to 4,908,059 without, and 363,210 adults with psoriasis matched to 1,801,875 without remained.

In the AE cohort, median follow-up was 5.5 years (IQR:2.2–10.4) in those with AE, and 4.8 years (IQR:1.8–9.6) in those without. In the psoriasis cohort, median follow-up was 5.9 years (IQR:2.3–11.5) in those with psoriasis, and 5.9 years (IQR:2.4–11.4) in those without. People with and without AE or psoriasis had broadly similar age, sex, and deprivation (Table 1 and Table S2). More than half of individuals in both cohorts had missing ethnicity data, therefore ethnicity was only considered in sensitivity analyses. Fewer adults with AE or psoriasis had missing BMI and smoking data. Those with missing BMI or smoking status were more likely to be young and male compared to those with complete data (Tables S3 and S4).

**Table 1:** Characteristics of Atopic Eczema and Psoriasis Cohorts at Cohort Entry

	Atopic Eczema Cohort		Psoriasis Cohort	
	With Atopic Eczema n=1,023,232	Without Atopic Eczema n=4,908,059	With Psoriasis n=363,210	Without Psoriasis n=1,801,875
**Follow-up^a^**				
Total person-years	6,995,892	30,935,170	2,680,394	13,246,255
Median (IQR) duration of follow-up (years)	5.5 (2.2–10.4)	4.8 (1.8–9.6)	5.9 (2.3–11.5)	5.9 (2.4–11.4)
**Sex**				
Female (%)	596,388 (58.3%)	2,842,125 (57.9%)	189,511 (52.2%)	940,489 (52.2%)
**Age (years)^b^**				
18–29	326,309 (31.9%)	1,566,148 (31.9%)	78,207 (21.5%)	389,718 (21.6%)
30–39	159,606 (15.6%)	796,251 (16.2%)	70,325 (19.4%)	349,056 (19.4%)
40–59	261,153 (25.5%)	1,274,882 (26.0%)	119,899 (33.0%)	593,805 (33.0%)
60+	276,164 (27.0%)	1,270,778 (25.9%)	94,779 (26.1%)	469,296 (26.0%)
**Quintiles of Carstairs deprivation index^c^**				
1 (least deprived)	128,266 (12.5%)	612,978 (12.5%)	43,233 (11.9%)	214,804 (11.9%)
2	202,153 (19.8%)	969,896 (19.8%)	68,391 (18.8%)	339,661 (18.9%)
3	211,882 (20.7%)	1,021,847 (20.8%)	76,487 (21.1%)	379,580 (21.1%)
4	247,290 (24.2%)	1,185,082 (24.1%)	88,207 (24.3%)	437,347 (24.3%)
5 (most deprived)	199,370 (19.5%)	954,675 (19.5%)	70,341 (19.4%)	348,450 (19.3%)
Missing	34,271 (3.3%)	163,581 (3.3%)	16,551 (4.6%)	82,033 (4.6%)
**Body mass index (kg/m^2^)^d^**				
Underweight (<18.5)	25,486 (2.5%)	122,658 (2.5%)	6,767 (1.9%)	37,208 (2.1%)
Normal (18.5–24.9)	368,313 (36.0%)	1,680,277 (34.2%)	118,438 (32.6%)	610,457 (33.9%)
Overweight (25–29.9)	274,062 (26.8%)	1,219,689 (24.9%)	106,417 (29.3%)	497,192 (27.6%)
Obese (30+)	181,183 (17.7%)	773,562 (15.8%)	77,511 (21.3%)	312,832 (17.4%)
Missing	174,188 (17.0%)	1,111,873 (22.7%)	54,077 (14.9%)	344,186 (19.1%)
**Smoking status^d^**				
Non-smoker	529,881 (51.8%)	2,433,856 (49.6%)	149,937 (41.3%)	866,540 (48.1%)
Current or ex-smoker	446,798 (43.7%)	2,006,409 (40.9%)	196,330 (54.1%)	788,823 (43.8%)
Missing	46,553 (4.5%)	467,794 (9.5%)	16,943 (4.7%)	146,512 (8.1%)
**Harmful alcohol use (%)^d^**	73,353 (7.2%)	294,033 (6.0%)	22,822 (6.3%)	89,889 (5.0%)
**Sleep problems (%)^d^**	278,516 (27.2%)	877,318 (17.9%)	n/a	n/a
**Ethnicity**				
White	387,082 (37.8%)	1,783,293 (36.3%)	150,087 (41.3%)	608,246 (33.8%)
South Asian	25,119 (2.5%)	97,603 (2.0%)	4799 (1.3%)	24,687 (1.4%)
Black	10,964 (1.1%)	54,725 (1.1%)	1071 (0.3%)	13,520 (0.8%)
Other	7797 (0.8%)	44,126 (0.9%)	1811 (0.5%)	11,397 (0.6%)
Mixed	4013 (0.4%)	18,864 (0.4%)	816 (0.2%)	4398 (0.2%)
Not stated or missing	588,257 (57.5%)	2,909,448 (59.3%)	204,626 (56.3%)	1,139,627 (63.2%)
**Charlson comorbidity index^d^**				
Low (0)	636,704 (62.2%)	3,605,319 (73.5%)	252,430 (69.5%)	1,308,427 (72.6%)
Moderate (1–2)	340,946 (33.3%)	1,103,589 (22.5%)	94,223 (25.9%)	421,230 (23.4%)
Severe (3 or more)	45,582 (4.5%)	199,151 (4.1%)	16,557 (4.6%)	72,218 (4.0%)

**Notes**: Values are numbers (percentages) unless otherwise stated. Individuals can contribute data as both atopic eczema or psoriasis exposed and unexposed. Therefore, numbers of exposed/unexposed do not total the whole cohort, as individuals may be included in more than one column. ^a^Follow-up based on censoring at the earliest of: death, no longer registered with practice, practice no longer contributing to CPRD, or severe mental illness diagnosis. ^b^Age at index date. ^c^Carstairs deprivation index based on practice-level data (from 2011). ^d^Based on records closest to index date.

**Abbreviation**: IQR, interquartile range.

### Main Analysis

After adjusting for matching variables (age, sex, general practice) and potential confounders (calendar period, deprivation), both AE and psoriasis were associated with at least a 17% increased hazard of SMI (AE: HR=1.17, 95% CI:1.12–1.22; psoriasis: HR=1.26, 95% CI:1.18–1.35) (Table 2). Small differences in absolute SMI risk were seen between individuals with and without AE or psoriasis (Table S5). After additionally adjusting for potential mediators (comorbidity burden, harmful alcohol use, smoking status, and BMI, and, in AE only, sleep problems and high-dose glucocorticoid use), associations with SMI did not persist in adults with AE (HR=0.98, 95% CI:0.93–1.04) and were attenuated in adults with psoriasis (HR=1.14, 95% CI:1.05–1.23). In sensitivity analyses, we saw similar results to the main analyses, however, additionally adjusting for ethnicity attenuated associations between AE or psoriasis and SMI (Table S1).

**Table 2:** HRs (95% CI) for the Association Between Atopic Eczema or Psoriasis and Severe Mental Illness. Fitted to Adults with Complete Data for All Variables Included in Each Model and from Valid Matched Sets^a^

Cohort	Minimally Adjusted			Confounder Adjusted^b^			Additionally Adjusted for Potential Mediators^c^		
	Number	Events/PYAR	HR (95% CI)^d^	Number	Events/PYAR	HR (95% CI)^d^	Number	Events/PYAR	HR (95% CI)^d^
**Atopic eczema**									
Unexposed	4,908,059	11,999/30,935,170	1 (reference)	4,744,478	11,428/29,565,265	1 (reference)	3,117,531	8,131/21,282,283	1 (reference)
Exposed	1,023,232	3,150/6,995,891	1.16 (1.12, 1.21)	988,961	3,012/6,686,453	1.17 (1.12, 1.22)	793,030	2,632/5,789,012	0.98 (0.93, 1.04)
**Psoriasis**									
Unexposed	1,801,875	4,598/13,246,255	1 (reference)	1,719,842	4,319/12,491,309	1 (reference)	1,179,789	3,252/9,176,327	1 (reference)
Exposed	363,210	1,191/2,680,394	1.27 (1.19, 1.36)	346,659	1,107/2,523,477	1.26 (1.18, 1.35)	286,396	990/2,218,106	1.14 (1.05, 1.23)

**Notes**: ^a^Matched sets including one exposed patient and at least one unexposed patient. ^b^Adjusted for calendar period and quintiles of Carstairs deprivation index (using 2011 census data). ^c^AE cohort is further adjusted for comorbidity burden (using the Charlson comorbidity index), sleep problems, smoking status, high dose glucocorticoid use, harmful alcohol use and body mass index. Psoriasis cohort is further adjusted for comorbidity burden (using the Charlson comorbidity index), smoking status, harmful alcohol use and body mass index. ^d^Estimated hazard ratios from Cox regression with current age as underlying timescale, stratified by matched set (matched on age at cohort entry, sex, general practice, and date at cohort entry).

**Abbreviations**: CI, confidence interval; HR, hazard ratio; PYAR, person-years at risk.

### Secondary Analysis

#### Skin Disease Severity

**Figure 3:**
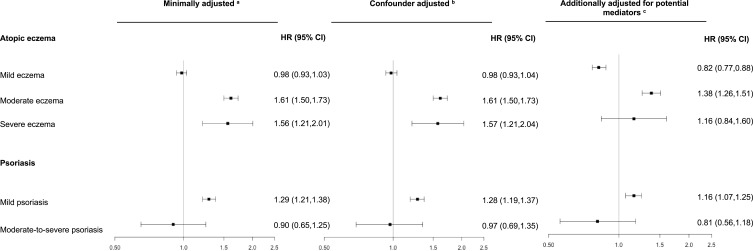
HRs (95% CI) for the association between atopic eczema or psoriasis severity and severe mental illness. Models fitted to adults with complete data for all variables included in each model and from valid matched sets. ^a^Model implicitly adjusted for matching variables. ^b^Model further adjusted for potential confounders (socioeconomic deprivation – using Carstairs index – and calendar time. ^c^Confounder adjusted model additionally adjusted for potential mediators (comorbidity burden [using Charlson comorbidity index], smoking status, harmful alcohol use, body mass index, and in atopic eczema analyses only, problems with sleep and high-dose glucocorticoid use).

In adults with severe AE and moderate-to-severe psoriasis, there were only a small number of SMI events (severe AE: 89 events; moderate-to-severe psoriasis: 43 events). We saw evidence that, compared to individuals without AE, individuals with moderate or severe AE had increased hazard of SMI (confounder-adjusted HRs: moderate AE: HR=1.61, 95% CI:1.50–1.73; severe AE: HR=1.56, 95% CI: 1.21–2.01) (Table S6, Figure 3). Compared to adults without psoriasis, individuals with mild psoriasis had increased hazard of SMIs (confounder-adjusted HR=1.28, 95% CI:1.19–1.37), while there was no evidence individuals with moderate-to-severe psoriasis were at increased risk of SMI (confounder-adjusted HR=0.97, 95% CI:0.69–1.35).

#### Effect Modification

We found no evidence the relationship between AE and SMI varied by sex or calendar period, but we found evidence of effect modification by age group (p<0.01). Adults aged 30–39 (HR=1.23, 95% CI:1.11–1.35) or 40–59 (HR=1.37, 95% CI:1.26–1.48) had the highest HRs for associations between AE and SMI (Table S7). In adults with psoriasis, we found no evidence associations with SMI varied by sex. However, we saw evidence (p=0.01) psoriasis was associated with increased SMI in all age groups apart from adults aged 30–39 (Table S7). We also saw evidence (p=0.01) of effect modification by calendar time, with HRs for associations between psoriasis and SMI higher in 2004–2009, 2010–2015, and 2016–2020 than in 1997–2003.

#### Individual Effects of Potential Mediators

Including sleep problems in our confounder-adjusted model reduced the HR for associations between AE and SMI from 1.17 (95% CI:1.12–1.22) to 0.98 (95% CI:0.94–1.02) (Table S8). Individual effects of other potential mediators on associations between AE or psoriasis and SMI were minimal.

## Discussion

After adjusting for confounders, AE and psoriasis were associated with a 17% and 26% increase respectively in the hazard of incident SMI compared to matched comparators. After further adjusting for potential mediators, associations with SMI did not persist in adults with AE and were attenuated in adults with psoriasis. SMI risk was highest in adults with moderate or severe AE and mild psoriasis, compared to those without AE or psoriasis.

### Strengths and Limitations

To our knowledge, this is the largest study to investigate temporal associations between AE or psoriasis and incident SMI in adults. The CPRD dataset used is broadly representative of the UK population,^28^ suggesting our results are generalisable to the UK population with AE or psoriasis. We identified adults with AE or psoriasis in primary care using validated definitions.^29^,^30^ We used DAGs to inform covariate selection.

However, our study has several limitations. Firstly, the definition used to identify AE (a validated definition requiring records of at least one diagnostic code, and at least two skin disease therapies)^29^ may introduce selection bias as it excludes untreated adults who may have milder disease. Additionally, not all adults will consult their general practitioner about their AE or psoriasis, meaning they could be incorrectly classified as not having AE or psoriasis. Misclassification of AE or psoriasis exposure in this study may be independent of SMI, biasing our estimates of associations between AE or psoriasis and SMI towards the null. Conversely, SMI ascertainment may be more likely in adults with AE or psoriasis due to increased contact with primary care because of their skin disease, leading to a potential overestimate of associations between AE or psoriasis and SMI. However, our sensitivity analysis restricting study inclusion to adults with at least one consultation in the year prior to cohort entry produced results like the main analyses of increased SMI in adults with AE and psoriasis.

Using CPRD data to capture SMI outcomes may miss individuals with SMI as the condition is usually identified in secondary mental health care. However, it is likely numbers of missed individuals with SMI will be small as in the UK, GPs have a central role in the care of people with SMI. Since the introduction of the Quality and Outcomes Framework (QOF) in 2004, GPs receive renumeration for the care of people with SMIs and maintaining a register of individuals with an SMI diagnosis.^40^

Our definition of AE or psoriasis severity may have misclassified adults with severe disease as having milder disease if they refused skin disease therapies, reducing numbers of individuals classified as having severe disease, and underpowering our analyses of associations between AE or psoriasis severity and SMI.

We may have introduced selection bias in our mediator-adjusted estimates, as we conducted complete case analyses. BMI and smoking data were more likely to be missing in adults without AE or psoriasis, and those with missing BMI or smoking data were more likely to be young and male. However, percentages of individuals excluded from analyses due to missing data are relatively low compared to the overall size of the cohorts; therefore, effects on our study are likely to be small.

We were unable to robustly capture all information on some potential confounders of associations between AE or psoriasis and SMI. For example, individual-level Carstairs deprivation data were only available for individuals in England, and practice-level data may not accurately represent individual-level deprivation. Additionally, information on potential mediators (eg, self and social stigmatisation due to visible skin disease, high levels of stress, substance misuse) of associations between AE or psoriasis and SMI were unavailable because they are either not captured, or captured incompletely, in routinely collected primary care data. Our estimates of associations between AE or psoriasis and SMI may therefore include residual effects of incompletely captured confounders and mediators.

Our cohorts included prevalent and incident AE or psoriasis, which is appropriate for chronic relapsing conditions like AE and psoriasis where exact onset date cannot be captured accurately using electronic health records.^41^ We captured some potential mediators (eg, comorbidity burden) on or before index date, and consequently some mediators may have occurred before eczema or psoriasis diagnosis (ie, not on the causal pathway after exposure as our analysis strategy assumes). However, given that approximately one-third of people with AE or psoriasis (31% AE and 37% psoriasis) entered cohorts on the date of first AE or psoriasis record, and eczema often starts in childhood,^1^ the timing of our capture of mediators in relation to AE or psoriasis onset may have limited effect on our mediator-adjusted estimates.

### Comparisons to Existing Literature

Our findings of increased risk of SMI in those with atopic eczema or psoriasis are consistent with limited longitudinal studies aiming to address temporal associations between atopic disorders or psoriasis and specific SMI outcomes.^23–27^ However, those studies were limited by 1) inclusion of children and adolescents in skin disease cohorts (due to known differences in psychiatric diagnostic practices between adults and children); 2) investigating atopic diseases in general, with a lack of focus on AE; 3) investigating specific SMIs such as schizophrenia, rather than SMI all together; () smaller study populations; and 5) inability to explore reasons for associations between AE or psoriasis and SMI due to adjustment for limited confounders and/or mediators. Additionally, previous longitudinal studies in the US used data from administrative health insurance databases, further limiting their ability to adjust for key covariates and may introduce selection bias (people with health insurance in the US may be different to people without).

Our study addresses some limitations of existing studies and adjusts for key confounders and mediators to investigate reasons for associations between AE or psoriasis and SMI. We found that associations between AE and SMI were strongly mediated by sleep problems. This finding is consistent with evidence from previous studies where AE disturbs sleep due to persistent itching,^42^ and disturbed sleep is linked to schizophrenia-like symptoms (eg, hallucinations and distorted perception) and can precede the onset of SMI.^19^,^43^

### Implications for Research and Clinical Practice

Our study, along with previous studies establishing temporal relationships between AE or psoriasis and other mental health conditions (ie, depression, anxiety),^10^,^44^ highlights the importance of monitoring mental health in adults with AE or psoriasis. Evidence suggests a large burden of psychological distress in adults with AE or psoriasis, and a lack of focus on mental health may negatively impact health of affected individuals. Unrecognised mental illness may reduce treatment adherence for skin conditions,^45^ lessening skin treatment benefits, potentially worsening the skin condition, and subsequently contributing to potentially worsening mental health. Recent UK guidelines suggest clinicians assess the impact of AE or psoriasis on the psychological wellbeing of those affected and use validated tools to objectively assess quality of life.^46^,^47^ However, evidence suggests individuals that present with physical symptoms (eg, AE or psoriasis) are less likely to have their mental health conditions detected.^48^ Introducing targeted mental health screening in UK primary care of adults with AE or psoriasis may allow early detection and intervention of mental health conditions. Improved identification of clinically significant depression and anxiety in people with psoriasis has been seen after introduction of mental health screening in specialist dermatology clinics.^49^

Our study also highlights the importance of identifying modifiable risk factors as they offer an opportunity to intervene and provide treatments that may reduce SMI risk. We found that associations between AE and SMI were largely mediated by sleep problems. Enhanced treatment of underlying skin disease using highly effective targeted therapies (ie, dupilumab) may have an additional effect of reducing sleep problems;^50^ however, additional strategies addressing insomnia with evidence-based therapies may also have a key role.^51^ Additional research should further investigate the mediating factors of associations between psoriasis and SMI.

## Conclusions

Adults with AE or psoriasis appear to be at increased risk of SMI compared to adults without AE or psoriasis. In adults with AE, and to a lesser extent in adults with psoriasis, increased risk may be explained by mediating factors (eg, sleep problems or lifestyle factors). Adults with mild psoriasis and more severe AE were at greater risk of SMI. Prevention strategies including targeted mental health screening and modifying mediating factors should be considered to reduce SMI burden in adults with AE or psoriasis.

## Abbreviations

AE, atopic eczema; BMI, body mass index; CI, confidence interval; CPRD, Clinical Practice Research Datalink, HR, hazard ratio; SMI, severe mental illness.

## Declarations

This paper was presented at the Society for Academic Primary Care (SAPC) Annual Scientific Meeting (ASM) 2022 as a short oral presentation. The abstract for the talk can be found on the SAPC ASM 2022 website (doi: https://sapc.ac.uk/doi/10.37361/asm.2022.1.1). This study is based in part on data from the Clinical Practice Research Datalink obtained under license from the UK Medicines and Healthcare products Regulatory Agency. The data are provided by patients and collected by the NHS as part of their care and support. The interpretation and conclusions contained in this study are those of the author/s alone.

## Data Sharing Statement

Data may be obtained from a third party and are not publicly available. Data from this study were obtained from the Clinical Practice Research Datalink (CPRD) and to guarantee patient data confidentiality, only the authors have access to the data during the study, and the data cannot be distributed to other parties. Data are available directly from CPRD subject to independent approval.
